# Smoking cessation and care management for veterans with posttraumatic stress disorder: a study protocol for a randomized controlled trial

**DOI:** 10.1186/s12913-015-0706-6

**Published:** 2015-02-01

**Authors:** Jamie Peterson, Allan V Prochazka, Catherine Battaglia

**Affiliations:** Department of Veterans Affairs, Eastern Colorado Health Care System, Denver, CO USA; University of Colorado, Aurora, CO USA

**Keywords:** Smoking cessation, Posttraumatic Stress Disorder, PTSD, Telehealth, Motivational interviewing, Veteran

## Abstract

**Background:**

Smoking remains the leading cause of preventable illness and mortality in the United States. Individuals with Posttraumatic Stress Disorder (PTSD) have smoking rates higher than that of others and fewer individuals with PTSD have quit smoking. This randomized controlled trial was designed to test the effectiveness of integrating telehealth care management and smoking cessation with motivational interviewing for Veterans with PTSD.

**Methods/Design:**

All smokers with PTSD, regardless of their desire to quit, were invited to participate. Enrollment occurred between November 2009 and April 2013. Target enrollment was 120 participants. Enrolled participants were randomized to either the control group, receiving usual care including a telehealth PTSD program, with a device that delivered PTSD information and in-home care management, or the intervention group, which included (1) a telehealth PTSD program, (2) motivational interviewing-based smoking cessation curricula via the telehealth device, and (3) weekly motivational interviewing counseling phone calls. Outcomes are self-reported 24-hour quit attempts, progression along the stages of change and 7-day point prevalence quit smoking rates for the intervention group compared to usual care alone. Secondary outcomes include participants’ perception of care coordination, patient satisfaction with motivational interviewing, PTSD symptoms, pain, depression and quality of life.

**Discussion:**

Motivational interviewing has been shown to increase readiness for change and smoking cessation care has been shown to be more successful when incorporated into in-person mental health care. Our study builds on previous studies. It integrates a written smoking cessation curriculum and phone-based motivational interviewing counseling into an established PTSD home telehealth care coordination program. This paper describes the design and methods of our randomized control trial.

**Trial registration:**

ClinicalTrials.gov, NCT00908882, May 22, 2009.

## Background

Individuals with Posttraumatic Stress Disorder (PTSD) who smoke represent a challenge to the Veterans Health Administration (VHA). Smoking remains an important cause of preventable illness and mortality [1] and is responsible for over 435,000 deaths per year [2]. Individuals with PTSD smoke at higher rates than those without mental illness [3] and only half as many individuals with PTSD have quit smoking as compared to those without mental illness [3]. As individuals experience reductions in all-cause mortality when they quit smoking, even a small increase in quit rates can improve health and life expectancy [4].

A bidirectional causal relationship is the likely cause for the difficulty individuals with PTSD have in quitting smoking [5]. On one hand smoking can increase PTSD symptoms such as hyperarousal and avoidance [6]. While on the other hand, nicotine can temporarily reduce anxiety and create a positive effect on mood [7]. This dichotomy can be most effectively addressed in mental health treatment, and integrating smoking cessation care into mental health treatment has been found to lead to longer periods of prolonged quitting [8]. However, tobacco cessation is not well integrated into ongoing mental health care. It tends to be brief and episodic, and lack of time during an appointment has been shown to be a major barrier for mental health providers in delivering smoking cessation counseling [9].

Although tobacco cessation programs are available in most VHA medical facilities, the rate of Veterans taking advantage of such programs has traditionally been low; only 17% of Veterans who are most interested in quitting participate in a smoking cessation program [10]. The distance that many Veterans live from medical centers contributes to the difficulty accessing health care. Most services are provided at tertiary care centers in major cities whereas approximately 28% of Veterans live in rural areas [11].

Home telehealth platforms with care coordination used by the VHA have been shown to decrease utilization of health care resources and have a mean satisfaction score rating of 86%, according to routine analysis of data from a cohort of 17,025 users [12]. It has also been shown to improve outcomes for high-risk and high-use individuals [13], such as those with PTSD. Since patients with PTSD tend to avoid activities and struggle to be around others [14], home telehealth can help these high-risk patients feel connected and less isolated. Integrating smoking cessation into a home telehealth program for patients with PTSD is an innovative alternative to incorporating smoking cessation services in mental health care.

Motivational interviewing (MI) is a behavioral counseling approach that has been shown to be effective in helping people abstain from negative behaviors by resolving ambivalence about behavior and increasing internal motivation [15]. MI has also been shown to increase smoking cessation [16] when compared with brief counseling or usual care [17]. This counseling style is typically done in verbal exchanges and has been shown to have greater effects when conducted in multiple sessions of greater than 20 minutes [17]. However, the currently tested method creates a barrier for integration into already busy mental health clinics as it requires significant time.

A pilot study testing fidelity and feasibility was completed prior to starting this trial. It showed that it was feasible to deliver home telehealth care management with high treatment fidelity [18]. Based on the results of this pilot study, we developed a randomized controlled trial (RCT) to facilitate smoking cessation in Veterans with PTSD. We integrated a written smoking cessation curriculum and telephone MI counseling into the established PTSD home telehealth care coordination program. This paper describes the design and methods of our RCT.

## Methods/Design

### Study design overview

**Figure 1:**
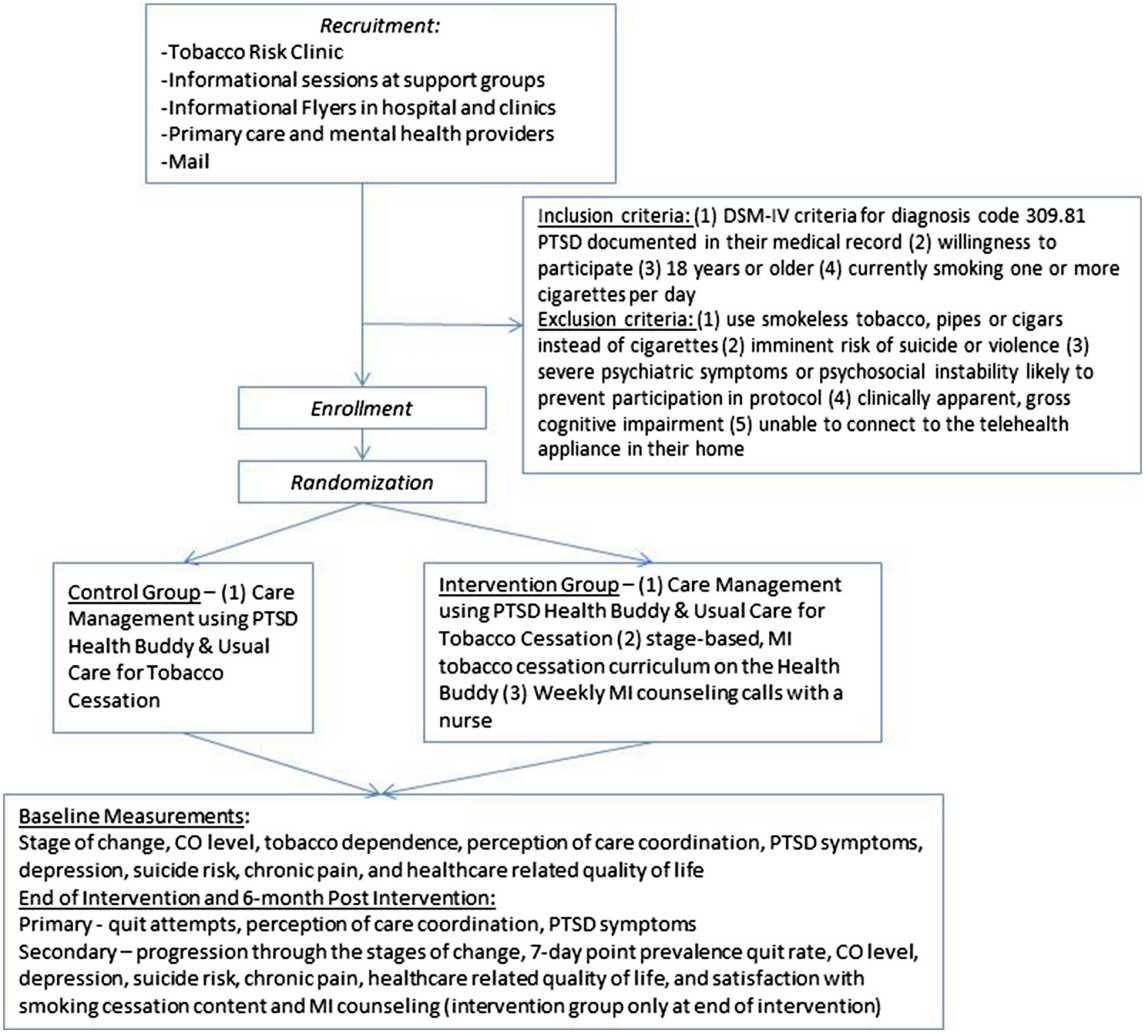
**Study design.**

This single center randomized trial is funded by the Department of Veterans Affairs Health Services Research and Development Service. It tests the effectiveness of integrated care management using telehealth and MI telephone counseling for smoking cessation treatment for Veterans with PTSD (Figure 1). A total of 120 participants were targeted for enrollment. Primary outcomes are self-reported quit attempts as measured by 24-hour point prevalence, perception of care coordination, and PTSD symptoms. Additional outcomes include progression along the stages of change and 7-day point prevalence quit rate. Outcome data will be measured at the end of the intervention period (month 3) and 6 months post-intervention (month 9). This study followed all applicable IRB regulations and was approved by the Colorado Multiple Institutional Review Board.

### Study population

All eligible smokers are offered enrollment, regardless of their desire to quit smoking.

Inclusion criteria include: Veterans that have a Diagnostic and Statistical Manual of Mental Disorders IV (DSM-IV) criteria for diagnosis code 309.81 PTSD documented in their medical record, willingness to participate, 18 years or older and currently smoking one or more cigarettes per day at the time of recruitment. Inclusion criteria initially included smoking ten or more cigarettes per day but was lowered to expand the pool of potential enrollees, while ensuring participation of daily smokers.

Exclusion criteria include: Veterans who use smokeless tobacco, pipes or cigars instead of cigarettes, have imminent risk of suicide or violence, have severe psychiatric symptoms or psychosocial instability likely to prevent participation in protocol (mental health or primary care provider will assess appropriateness), have clinically apparent, gross cognitive impairment, do not have telephone access and/or are unable to connect to the telehealth appliance in their home.

#### Control group

Participants randomized to the control group will receive information programmed into the usual PTSD Health Buddy program, care coordination and usual care for smoking cessation.

#### Intervention group

Participants randomized to the intervention group will receive (1) information programmed into the usual PTSD Health Buddy program, care coordination and usual care for smoking cessation (2) stage-based, MI smoking cessation curriculum on the Health Buddy, and (3) weekly telephone MI counseling with a nurse.

### Recruitment

Written consent to participate is obtained. Recruitment occurred between November 2009 and April 2013. Recruitment is performed by creating and posting an informational flyer in high traffic areas in the VHA hospital and community-based outpatient clinics. It is geared toward potential patients and explains goals and objectives of the study, eligibility criteria and enrollment process. Patients are also approached in the VHA Tobacco Risk Clinic and VHA mental health and smoking support groups to offer participation. Additionally, primary care and mental health providers are educated about the study and their assistance in identifying eligible patients is enlisted.

Recruitment is also performed utilizing Electronic Medical Record screening and the mail. Names, addresses, and primary care provider of potential subjects from the VHA database who have the ICD-9 codes 309.81 (PTSD) and 305.1 (Tobacco Use Disorder) in their medical record are identified. An invitation to participate in the study, an information sheet, a questionnaire and a refusal card are sent addressed from the primary care provider via the U.S. Mail. If the potential subject returns the questionnaire, he/she will be contacted for possible enrollment by a member of the research team. If the potential subject returns the refusal postcard, the subject’s name is removed. If no response is received, the potential subject will receive up to two more reminder letters. If no response is received after three attempts, the subject’s name will be removed from the database.

### Intervention

**Figure 2:**
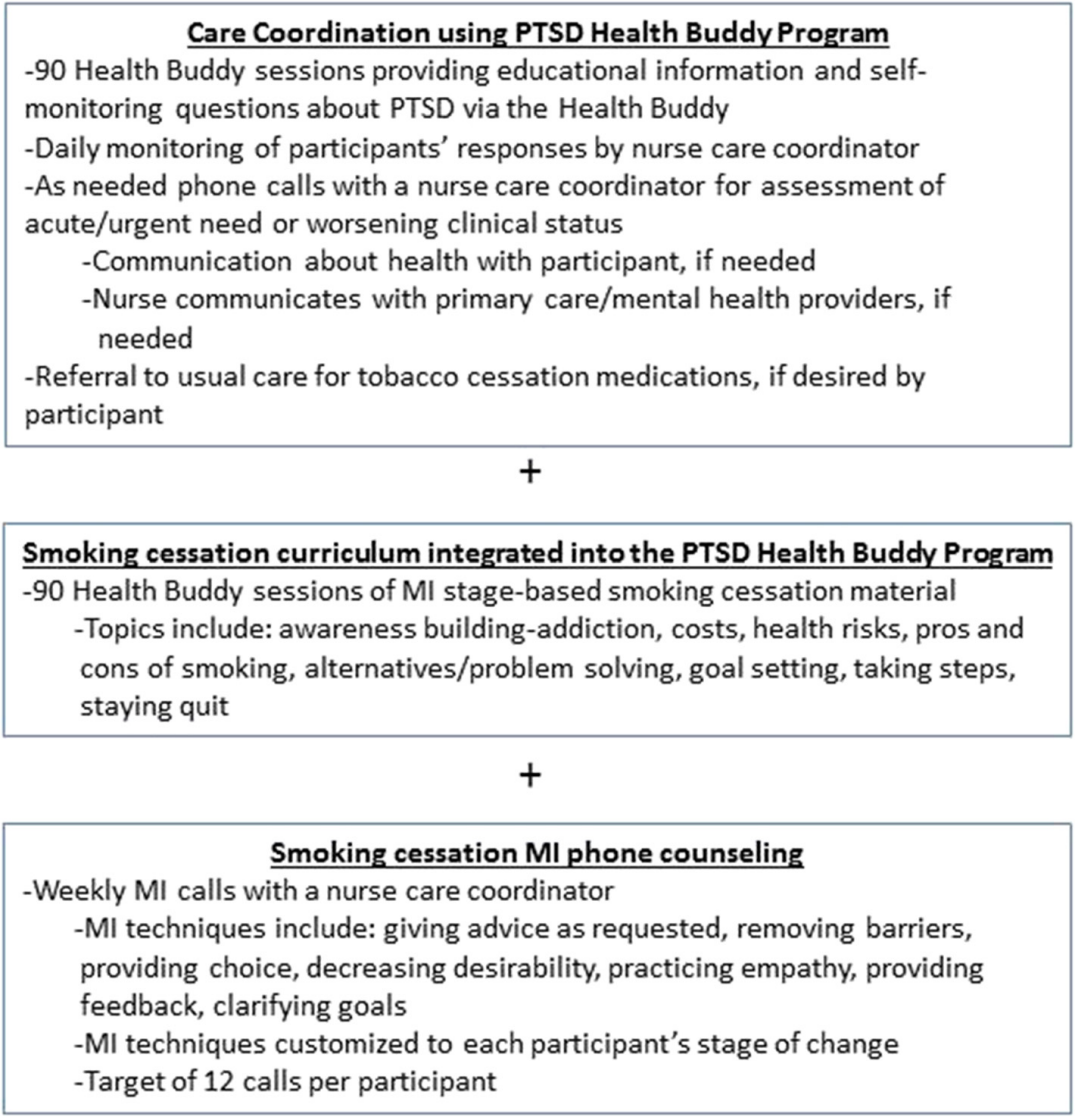
**Intervention components.**

All participants receive the PTSD home telehealth program during the intervention portion of the study. Only the intervention group receives smoking cessation information via home telehealth and MI counseling by a nurse (Figure 2). The care coordinator working with the intervention group is trained in MI, whereas the care coordinator working with the control group provides usual care.

**Figure 3:**
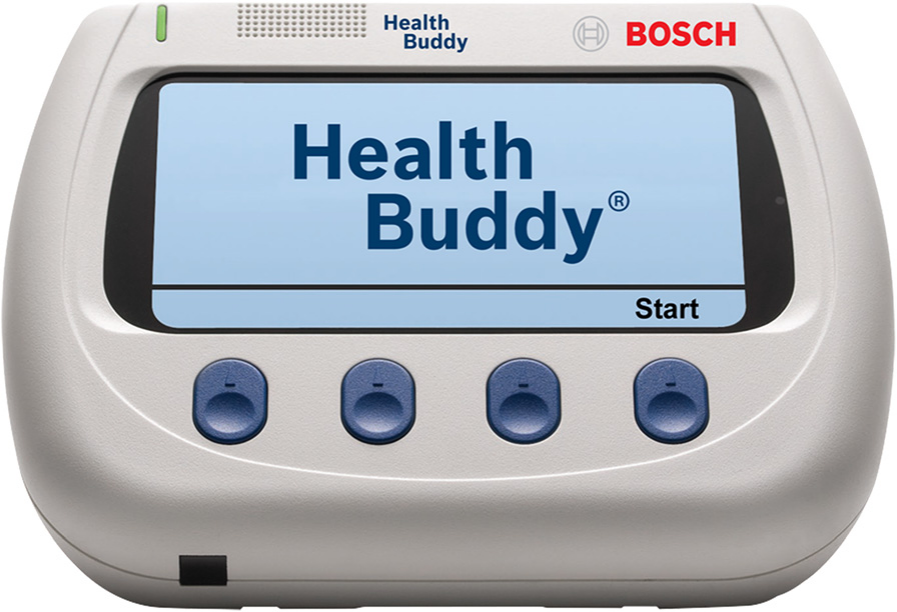
**Health Buddy device.**

Home telehealth, as provided via the Health Buddy® appliance (Bosch Healthcare, Palo Alto, CA), is a chronic disease management tool used by the VHA (Figure 3). The Health Buddy is a 12 inch × 8 inch × 4 inch computer-like appliance, consisting of a LCD screen and four large buttons, used by patients to enter responses. Individuals use the Health Buddy daily from their home, answering questions and reading messages that are displayed on the screen. A typical session takes one to three minutes to complete. The device’s questions aim to assess symptoms, behaviors and knowledge related to an individual’s PTSD. An example of a question in the PTSD module is “On a scale from 1–10, please report your highest anger rating in the past three days”. The device automatically transmits the patients’ responses during the night to a secure data center and questions for the next day are downloaded into the appliance.

A nurse care coordinator reads responses daily, monitoring for gradual changes in individuals’ status and intervening only in acute or urgent care needs. Responses are color-coded and flagged according to pre-determined criteria to facilitate rapid interpretation. As all responses for a given individual are summarized, the care coordinator can determine at a glance whether or not a person is experiencing significant problems. When deterioration in clinical status is detected, the care coordinator will contact the individual via telephone to verify the accuracy of responses and acquire further information. Depending on the responses, the care coordinator will either provide counseling or discuss the person with the primary care or mental health provider for subsequent recommendations. Data are accessed by logging onto a secure VHA intranet site to review responses daily during the regular work week.

The Health Buddy home telehealth intervention aims to promote self-care management which is a pillar of the Chronic Care Model [19,20]. It gives patients with chronic conditions support and guidance in order to encourage daily decisions that improve health-related behaviors and clinical outcomes. Self-management support is patient-centered and offers patients a variety of tools or techniques that help them choose healthy behaviors and transforms the patient-caregiver relationship into a collaborative partnership [21]. Through the PTSD Health Buddy, the patient will receive information about managing his/her multiple chronic conditions that foster self-management.

**Figure 4:**
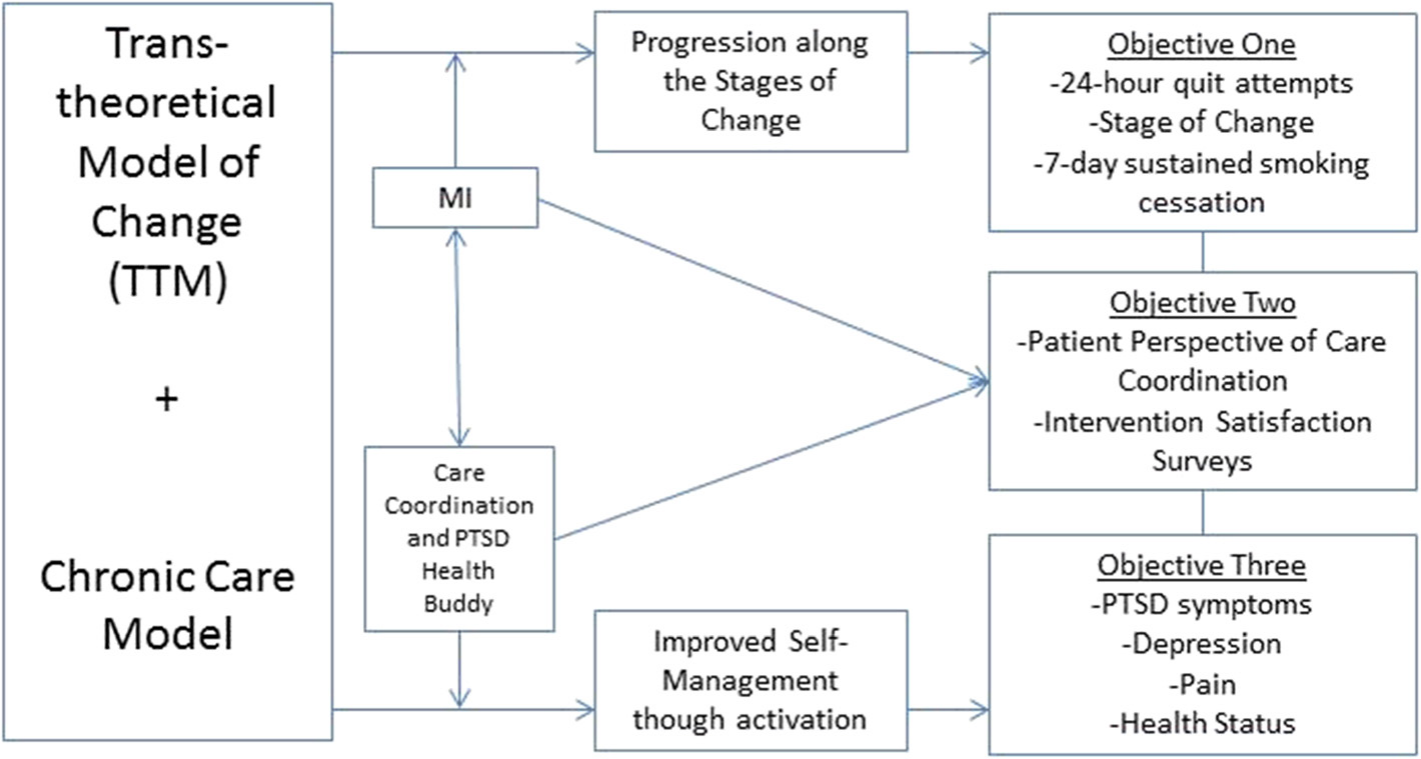
**Conceptual model.**

Integrated within the PTSD Health Buddy, the intervention utilizes the Transtheoretical Model of Change (TTM) as the theoretical framework on which to structure the MI curriculum and counseling. TTM [22,23] is based on the principle that individuals are at various stages of readiness to make a behavior change, like quitting smoking. As individuals progress towards making a change to a behavior, they move along categories referred to as the stages of change. Our conceptual model (Figure 4) is based on both the Chronic Care Model and the TTM. We hypothesize behavior change will result from strengthening self-management skills and enhancing motivation to move along the stages of change.

Participants are asked to use the research Health Buddy daily for 90 sessions (the intervention period). During the follow up period (final six months), monthly calls are made by the research staff to gather smoking data. If any participant wants to continue to use the Health Buddy after the intervention period, the individual’s telehealth care is transferred to the VHA’s Care Coordination Home Telehealth Department and they use the standard PTSD Health Buddy program.

All participants have access to usual tobacco cessation care at the VHA, which includes a specialty tobacco cessation clinic, a tobacco cessation support group in the mental health clinic, a month long tobacco cessation educational group, and/or treatment within the primary care setting. All first line medications are available to study participants, including bupropion SR, nicotine gum, nicotine lozenge, nicotine patch. Varenicline, nicotine nasal spray and nicotine inhaler use is restricted by the VHA for use by those who failed other medication therapies. In order to utilize existing treatment modalities, study nurses assess participants’ desire to use a medication and, if interested, a referral is made to existing providers.

Consistent with MI principles, participants will make choices about how and when to participate. We acknowledge that not all participants will chose to answer the Health Buddy questions daily or to be available for their counseling call weekly.

#### Smoking cessation curriculum

The research team developed a 90-session smoking cessation curriculum based on the principles of TTM and MI that is integrated with the PTSD curricula. Using branching-chain logic, each session provides stage-based information or questions about readiness to quit smoking, process of change and a commitment to take action. An example of a question is “On a scale of 1 to 10 (with 1 being not confident and 10 being very confident), how confident are you that you are capable of quitting?”

#### Motivational interviewing counseling

The intervention group will participate in weekly counseling calls. Interactions with the research nurse are based on stage of change and responses to the home telehealth smoking cessation curriculum. MI techniques such as 1) giving advice as requested, 2) removing barriers, 3) providing choice, 4) decreasing desirability, 5) practicing empathy, 6) providing feedback, 7) clarifying goals, and 8) actively helping [15,24] are utilized. MI techniques will vary based on stage of change. For example, the individual in pre-contemplation is not currently considering a change. The care coordinator will validate lack of readiness, respect autonomy, encourage re-evaluation, and ask permission when explaining risks.

To ensure fidelity, random calls made by the intervention research nurse are observed throughout the study. Additionally, the research nurse will participate in ongoing MI training and workshops throughout the study to diminish “drift” away from the principles of MI.

### Outcome measures

The study has three objectives; each has a primary outcome and secondary endpoints. Secondary outcomes are exploratory and supportive of the primary outcomes.

In objective one, we will compare the (1) proportion of individuals that make a self-reported 24-hour quit attempt, (2) progression through the stages of change [25,26] and (3) 7-day point prevalence quit smoking rates for the intervention group to usual care alone. The primary outcome is the proportion of participants making a self-reported quit attempt in each group during the study period. Secondary outcomes are the proportions of individuals who make a progression in their stage of change and quit smoking, as measured by a 7-day point prevalence, between study entry, end of intervention (month 3) and at 6 months post-intervention (month 9). Quit attempts will be bioverified by expired carbon monoxide levels (a CO level of less than or equal to ten will be considered non-smoking) [27] during in-person visits at the 3 month and 6 month follow-up periods. Subjects who do not respond will be classified as continuing smokers.

In objective two, we will compare the patient perception of care coordination for the intervention group to usual care alone. The primary outcome is the mean score change of the participant’s perception of care coordination (PACIC) [28,29] from baseline to end of intervention (month 3), and 6 months post-intervention (month 9) periods. Secondary endpoints of interest are an evaluation of the intervention from the participants’ perspective via two satisfaction surveys (one for stage-based/MI smoking cessation Health Buddy written content and one for the nurse-driven MI intervention). Patient satisfaction will be evaluated for the intervention group only at the end of the intervention (month 3).

In objective three, we will compare PTSD symptoms of participants who receive the intervention to those receiving usual care alone. The primary outcome is the mean score change of PTSD symptoms utilizing the Posttraumatic Stress Disorder Checklist [30] from baseline to end of intervention (month 3), and 6 months post-intervention (month 9). Secondary endpoints of interest are the mean score change for the following survey results and will be compared from baseline to end of intervention (month 3) and 6 months post-intervention (month 9):

- Depression: Change in depressive symptoms measured by the Geriatric Depression Screen (GDS) [31,32]
- Chronic Pain: Change in chronic pain symptoms measured by the McGill Short Form Pain Questionnaire [33]
- Health Status: Change in the Health Care Related Quality of Life measured by the Veterans Rand-12 (VR-12) [34,35]

Sample size calculations were based on testing the effectiveness of the intervention, compared to the control group, in higher rate of quit attempts and progression in the stage of change, better perceived care coordination, and improved PTSD symptoms. Only the trend of quit rate differences will be examined since the study sample will include those who are not ready to quit. Initially we expected to enroll 140 subjects in order to obtain 120 completers, anticipating a 12-15% drop out rate. However, due to a higher than expected loss to follow up rate enrollment was expanded to target 180 participants. Statistical analyses will be done using SAS (Cary, NC). Objective one involves comparison of two proportions for self-reported quit attempt rate and stage change rate. With 120 subjects, equally allocated between the two groups, 24% absolute difference in rates at 3 months can be detected for a two-sided test of proportions between two groups at a 5% Type I error rate and 80% power. Objective two and three involve comparison of means across two groups for perceived care coordination and PTSD symptoms. Effect size of 0.1, 0.25 and 0.4 (or equivalently mean difference of 0.2, 0.5, 0.8 in standard deviation units between two groups) are typically defined as small, medium, and large. By recruiting the 120 subjects for objective one, a medium effect size in perception of care coordination and PTSD symptoms can be detected for a two-sided test of means between two groups at a 5% Type I error rate and 80% power. We will consider this finding to be clinically significant if intervention group Veterans perceive their care to be better coordinated and they experienced less PTSD symptoms.

### Data collection

Outcomes are assessed at the end of the intervention period (month three) and six months post-intervention (month 9). Data collection occurs at baseline, at set points during the intervention period, at the end of the intervention period (month 3), at set points during the follow-up period, and six months post-intervention (month 9). Many survey questions are entered into the Health Buddy program and then answered by the participants via the appliance. Each subject will attend an in-person study visit at designated times to complete surveys not able to be performed via the Health Buddy. A small incentive will be given to subjects (total of $24 for three visits) to help defray associated costs.

Baseline data include expired carbon monoxide level, McGill Pain Questionnaire (both done at in-person enrollment), PACIC, PTSD Checklist, Stage of Change, Fagerström Test of Nicotine Dependence [36], GDS, and VR-12. As these surveys, except when noted, are implemented via the Health Buddy and done at home by the participant, not all baseline survey results are recorded on the day of enrollment.

During the intervention period, a 24-hour cigarette recall is asked via the Health Buddy weekly. Stage of change and suicide risk are assessed monthly via the Health Buddy during the intervention portion of the study. At the end of the intervention period, the following data are collected: expired carbon monoxide level, McGill Pain Questionnaire (both done in person), PACIC, PTSD Checklist, Stage of Change, GDS, and VR-12. Participant satisfaction with the MI written content and the MI counseling calls is assessed at this time, as well. Unless otherwise noted, surveys are completed on the Health Buddy and therefore all participants do not complete them on the same day; it is dependent on how many days a week the Health Buddy is used by a participant.

During the six month follow-up period, stage of change and a 24-hour cigarette recall are asked monthly. At the end of the six-month follow-up, participation in the study ends and the following additional data are collected: expired carbon monoxide level, McGill Pain Questionnaire (both done in person), PACIC, PTSD Checklist, Stage of Change, GDS, and VR-12.

### Data analysis

All analyses will be conducted using the intention to treat principle. We will describe baseline characteristics by study groups and test for differences in age, gender, PTSD symptoms, depressive symptoms, suicide risk, chronic pain, medical co-morbidities, co-morbid mental health disorders and other patient attributes. If noted, we will account for significant differences in the analyses.

Analysis of objective one, primary outcome: We will test the primary hypothesis by comparing dichotomous variables, self-reported quit attempts as the outcome variable at the end of the intervention period (3 months) and the end of the follow-up period (6 months post-intervention) between the two study arms using chi-square tests. Then, to account for potential imbalance in baseline characteristics and to improve the precision of estimates, we will test the primary hypothesis using a logistic regression model with quit attempts as the outcome variable and include baseline characteristics, tobacco dependence, selected baseline variables, and study group as independent variables.

Analysis of objective one, secondary outcomes: Changes in readiness stages and smoking cessation will be compared between arms using chi-square tests. Then, to improve the precision of estimates, we will test the secondary outcomes using a logistic regression model. This model will use the changes in stage of change and smoking cessation as the dependent variables and baseline characteristics, tobacco dependence, selected baseline variables, and study group as independent variables.

Analysis of objectives two and three, primary outcomes: We will test the primary objectives for objectives two and three by comparing means (or changes in means) of continuous variables, patient’s perception of care coordination and PTSD symptoms at the end of the intervention period (3 months) and the end of the follow-up period (6 months post-intervention) between the two study arms using the Student’s t-test. Then, to account for any imbalance in baseline characteristics and to improve the precision of estimates, we will test the primary objectives of these hypotheses using a multiple linear regression model. This model will use the perception of care coordination primary outcome as the dependent variable (and PTSD symptoms) and include baseline characteristics, tobacco dependence, selected baseline variables, and study group as independent variables.

Analysis of outcomes for objectives two and three, secondary outcomes: Mean scores (or changes in the mean scores) of standardized instruments will be used to assess depression, suicide risk, chronic pain, and health related quality of life in all subjects. These are all continuous outcomes so we will use the t-tests and linear regression to determine intervention effects.

## Discussion

This study incorporates a written and verbal MI-based intervention by a nurse into a currently used home telehealth care coordination platform for Veterans with PTSD who smoke. We feel this program is unique and important for several reasons. One, it has the potential to facilitate utilization through increased access to smoking cessation care via use of telehealth and phone calls. This expands reach to Veterans who live at a significant distance from tertiary care centers or have difficulty obtaining clinic services due to PTSD symptoms or stigma. Two, it can potentially increase smoking cessation rates through incorporation of a smoking cessation program into a currently established mental health service for individuals with PTSD. Three, it increases the dose of the intervention without significant extra cost or time by using daily dosed written curricula. Finally, it tests the impact of a nurse-implemented MI counseling intervention.

This intervention attempts to alleviate barriers Veterans with PTSD face when accessing smoking cessation treatment. Its strength lies in its ability to provide smoking cessation and mental health care services via telehealth and the telephone. Providing care over the telephone is one way to increase availability of services. This intervention thereby facilitates the ease of interaction by providing in-home services, which decreases monetary and time resources spent traveling to a clinic for care. It also reduces potential stigma experienced when entering a VHA mental health clinic. Furthermore, it provides an opportunity for those living in very rural areas to utilize previously inaccessible smoking cessation treatment.

As smoking cessation interventions have been shown to be more successful when integrated into face-to-face mental health care visits [8], this intervention was designed to be *easily* implemented into current home telemonitoring mental health care. It is a comprehensive and intense approach that provides both mental health and smoking cessation services to Veterans with PTSD daily via the home telehealth platform, through weekly scheduled MI counseling calls, and, as needed, for immediate mental health issues and/or changes. It further tests the efficacy of the incorporation of smoking cessation care into mental health services by testing the delivery of an intervention over the phone and via telehealth. The Health Buddy platform is currently used nationally in the VHA for many diagnoses, including chronic obstructive pulmonary disease, congestive heart failure, diabetes and depression. It is a large chronic disease management initiative for the VHA; it was introduced in 2003 and aimed to have 92,000 enrollees by the end of 2012 [37]. Smoking cessation curricula could be easily added to any currently offered disease management program, and thus could reach many smokers quickly and easily. If shown to be effective, the ease of implementation of this program into currently in-use, established medical care is a strength of this intervention.

To our knowledge, limited studies have examined the effect of print-based MI interventions on behavior change. For this study, the research team wrote a smoking cessation curriculum using the tenants of MI. Smoking cessation assistance is administered via the telehealth appliance to the participant every day the Health Buddy is used for 90 sessions. Thus, the participant receives smoking cessation information more frequently than is typically administered in a class or counseling session. If successful, this method is a cost effective way to deliver frequent smoking cessation information to a large number of participants.

Nurses are ideal implementers because they have experience in teaching and increasing self-management and positive behaviors and have been shown to successfully manage chronic diseases via telehealth [38,39]. They have also been shown to improve health outcomes through the use of counseling and motivational approaches [18,40]. Additionally, because all care is telephone-based, the nurse is not bound by appointment times, allowing for more flexibility depending on individual needs of the patient. The average length of every phone call is recorded in the study. Ability of the care coordinators to deliver the intervention with high fidelity was tested and validated in the pilot study [18].

Another unique aspect of the study is its less restrictive inclusion criteria which increases its generalizability. All smokers, regardless of their desire to quit smoking are invited to participate, resulting in the potential to provide smoking cessation assistance to those who might not otherwise receive it. PTSD is often not a solitary diagnosis; 88% of men and 78% of women with PTSD also have at least one other mental health diagnosis [41]. Whereas some trials examining PTSD and smoking have excluded those with bipolar, substance abuse and schizophrenia, this study does not make a blanket statement to omit participants with those diagnoses. Ability to participate in a protocol, as decided by their current primary care or mental health provider, will allow participants with managed concurrent mental health diagnoses to participate. We loosened our inclusion criteria during the trial in order include participants who were smoking less than ten cigarettes a day. This change occurred to be more inclusive to interested participants. Thus, if successful, the results can be applied inclusively to a large population.

In conclusion, this study’s significance lies in its ability to test the incorporation of written and telephone MI based smoking cessation into an already established and utilized mental health care coordination program.

## Abbreviations

PTSD: Posttraumatic Stress Disorder
MI: Motivational interviewing
PACIC: Participant’s perception of care coordination
GDS: Geriatric depression scale
VR-12: Veterans Rand-12
